# Specific Entrainment of Mitral Cells during Gamma Oscillation in the Rat Olfactory Bulb

**DOI:** 10.1371/journal.pcbi.1000551

**Published:** 2009-10-30

**Authors:** François O. David, Etienne Hugues, Tristan Cenier, Nicolas Fourcaud-Trocmé, Nathalie Buonviso

**Affiliations:** 1 Neurosciences Sensorielles, Comportement, Cognition, CNRS–Université Claude Bernard, Lyon, France; 2 Department of Information and Communication Technologies, Universitat Pompeu Fabra, Barcelona, Spain; 3 IFR 19, Institut Fédératif des Neurosciences de Lyon, Lyon, France; RIKEN Brain Science Institute, Japan

## Abstract

Local field potential (LFP) oscillations are often accompanied by synchronization of activity within a widespread cerebral area. Thus, the LFP and neuronal coherence appear to be the result of a common mechanism that underlies neuronal assembly formation. We used the olfactory bulb as a model to investigate: (1) the extent to which unitary dynamics and LFP oscillations can be correlated and (2) the precision with which a model of the hypothesized underlying mechanisms can accurately explain the experimental data. For this purpose, we analyzed simultaneous recordings of mitral cell (MC) activity and LFPs in anesthetized and freely breathing rats in response to odorant stimulation. Spike trains were found to be phase-locked to the gamma oscillation at specific firing rates and to form odor-specific temporal patterns. The use of a conductance-based MC model driven by an approximately balanced excitatory-inhibitory input conductance and a relatively small inhibitory conductance that oscillated at the gamma frequency allowed us to provide one explanation of the experimental data via a mode-locking mechanism. This work sheds light on the way network and intrinsic MC properties participate in the locking of MCs to the gamma oscillation in a realistic physiological context and may result in a particular time-locked assembly. Finally, we discuss how a self-synchronization process with such entrainment properties can explain, under experimental conditions: (1) why the gamma bursts emerge transiently with a maximal amplitude position relative to the stimulus time course; (2) why the oscillations are prominent at a specific gamma frequency; and (3) why the oscillation amplitude depends on specific stimulus properties. We also discuss information processing and functional consequences derived from this mechanism.

## Author Summary

Olfactory function relies on a chain of neural relays that extends from the periphery to the central nervous system and implies neural activity with various timescales. A central question in neuroscience is how information is encoded by the neural activity. In the mammalian olfactory bulb, local neural activity oscillations in the 40–80 Hz range (gamma) may influence the timing of individual neuron activities such that olfactory information may be encoded in this way. In this study, we first characterize in vivo the detailed activity of individual neurons relative to the oscillation and find that, depending on their state, neurons can exhibit periodic activity patterns. We also find, at least qualitatively, a relation between this activity and a particular odor. This is reminiscent of general physical phenomena—the entrainment by an oscillation—and to verify this hypothesis, in a second phase, we build a biologically realistic model mimicking these in vivo conditions. Our model confirms quantitatively this hypothesis and reveals that entrainment is maximal in the gamma range. Taken together, our results suggest that the neuronal activity may be specifically formatted in time during the gamma oscillation in such a way that it could, at this stage, encode the odor.

## Introduction

Recent experiments under awake conditions indicate that there is little effective spatial contrast in the firing rate of neurons in olfactory structures [1] and even in auditory primary sensory structures [2], which suggests a limited role for the mean firing rate in sensory function. In addition, it has recently been shown in the retina that the sole firing rate is not sufficient to code for behavioral performance [3]. Furthermore, the role of spike timing for plasticity [4] and coding [5] suggests that the temporal structure of neuronal activity may be crucial for perception. In particular, various functional studies have reported that fast local field potential (LFP) oscillations, particularly those in the gamma band (40–80 Hz) that correlate with perception [6] and attention [7], simultaneously induce a greater synchrony in the firing of cells, thereby having a greater impact on downstream structures [7].

In the mammalian olfactory bulb (OB), odorant stimulation induces LFP oscillations both in the gamma (40–80 Hz) and beta (15–35 Hz) ranges. There is, however, significant disparity in the reports regarding the conditions in which these oscillations are expressed. In anesthetized animals, gamma and beta LFP oscillations are odor-induced and appear alternately along the respiratory cycle, with gamma bursts specifically occurring during the inspiration/expiration (I/E) transition [8],[9]. In the awake rat, gamma and beta LFP oscillations exist spontaneously, and odors evoke increases [10] or decreases [11] of amplitude in the gamma frequency range. In insects [5],[12] and to a lesser extent in fish [13], spiking activity has been shown to be strongly linked to the oscillation which plays therefore a crucial role in coding. In rodents the role of these oscillation has been primarily described to reflect the experience of the animal [14],[15]. Despite the findings of phase/time relationships between mitral (MC) spiking activity and the oscillation or between MC pairs [16]–[18], a better description of these relationships across the MC population is needed before demonstrating that such temporal activity can support an odor code.

Our approach focused on gamma oscillations in anesthetized rats, which are not very different from oscillations recorded under behaving conditions [19]. The aim of the current study was two-fold: (1) to analyze the fine temporal relationships between the spiking of an MC population and gamma oscillations in anesthetized, freely-breathing rats in response to odorant stimulation and (2) to build a biophysical model of MC activity under the same in vivo conditions to explain the data. Our data showed that during the transient periods of gamma oscillation, a specific subset of MCs exhibited specific periodic firing phase patterns that could be grouped in a small number of types. Furthermore, the pattern types exhibited by a particular MC are qualitatively related to the nature of the odorant stimulus. These different types of patterns constitute the signature of an entrainment of these cells by an input oscillation [20],[21], whose origin can be attributed to the inhibitory granular input [17]. In the model, we verify that this scenario of entrainment effectively reproduces the experimental observations. We further explored the model's dependence on MC input activity and intrinsic properties, showing that our model robustly exhibited an optimum of entrainment when the oscillation frequency was within the gamma range. Overall, these results provide a detailed quantitative description of the complex MC firing activity at the population level and, at the same time, a theoretical explanation of the links between MC spike trains and gamma LFP oscillations evoked under freely breathing conditions in the OB. Furthermore, these results constitute an important underlying mechanism if future research shows that such a firing activity can support odor coding in behaving conditions.

## Results

### Part I: experimental data analysis

#### Gamma LFP oscillations are recurrent and stable, and the firing rate is highly variable

**Figure 1:**
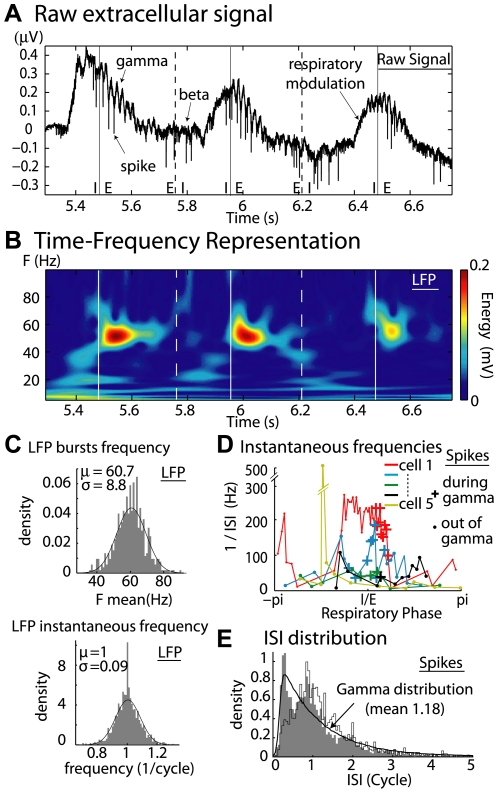
Unit activities and LFP gamma oscillation frequencies had the same mean but very different variability. A) Raw signal recorded in the MC layer during odorant stimulation. Vertical continuous lines: I/E transitions; vertical dashed lines: E/I transitions. B) Time frequency representation extracted from the raw signal and based upon a wavelet transformation. The signal energy at each time-frequency point is in a colorscale. C) *Top)* Distribution of LFP gamma-burst mean frequencies with a normal function fit, which is shown as a black trace. *Bottom)* Distribution of LFP cycle instantaneous frequency, which is defined as the inverse of the period between two max of the oscillation, and which is normalized to the average frequency of the gamma burst to which the cycle belongs. D) Instantaneous frequencies along one respiratory cycle (six colors for six cells). + are spike positions for which gamma LFP oscillations are present. E) Interspike interval distribution (ISI) for spike trains occurring during gamma oscillations (bin 0.05 cycle, *black line*, gamma function distribution with the same mean ISI as the experimentally found (see methods)). Contour histogram is the distribution of the mean ISIs that was calculated for each spike train. All units are given in “cycle”.

Under our anesthetized conditions, odorant stimulation elicited LFP oscillations in the whole OB and spiking activity in the MC layer. Respiratory modulation appeared as a slow, large-amplitude oscillation (∼2 Hz) that was visible on the raw trace (Fig. 1A). Although slower than under waking conditions, this respiratory modulation could be associated with theta range frequencies (4–10 Hz). The time-frequency representation (TFR) (Fig. 1B) led to the observation of two other oscillatory frequencies, namely beta oscillations in the 15–35 Hz range and gamma oscillations in the 40–80 Hz range. Gamma bursts recurred at each respiratory cycle around the inspiration/expiration (I/E) transition [8] (Fig. 1B) and were nearly independent of the odorant stimulus. In contrast, the characteristics of the beta bursts depended on the molecular features of the odor [9]. The gamma oscillation positions during the respiratory cycles were remarkably stable between odors, always starting at the I/E transition. The distribution of gamma burst mean frequencies was fitted with a narrow Gaussian distribution with a mean of 60.7 Hz (standard deviation: 8.8 Hz, see Fig. 1C, top). Inside each gamma burst, the cycle to cycle frequency was even more stable. Indeed when the frequency of each cycle (the inverse of its length) was normalized relative to the mean frequency of its gamma bursts, the resulting distribution could be fitted with a Gaussian distribution with a standard deviation that was lower than 0.1, which indicated low variability (Fig. 1C, bottom).

In contrast, the MC instantaneous frequencies were highly variable (i) within the same cell, between different epochs of the respiratory cycle, (ii) within the same cell, between odors, and (iii) between simultaneously recorded units. An example is given in Fig. 1D, which shows the course of instantaneous frequencies over the period of a respiratory cycle for six cells that responded to the same odor. These examples show that the MC activities are highly modulated and variable during the respiratory cycles, as previously described [22]–[24].

Due to the bulbar-specific origins of gamma oscillations and to the regular occurrence of these oscillations across the bulbar surface, we focused our measurements on neuron discharges during LFP gamma bursts. To simplify the comparison between bursts of different frequencies, the interspike intervals (ISI) will always be expressed in fractions of their gamma burst average period (the Cycle unit), and similarly, the MC spike rates will be expressed in spikes per cycle (SpC). The ISI distribution that was observed during these gamma bursts was plotted in Fig. 1E (grey bars). We compared these distributions with a gamma distribution (black line) that reflected the usual random discharge of a neuron with a similar refractory period (0.18 cycles ( = 3 ms)) and the same mean ISI (1.18 cycles) as the experimental distribution. We observed two major deviations of the experimental ISI distribution from the gamma distribution. One appears around 0.1–0.2 cycles and corresponds to the numerous spike doublets (or high frequency bursts) that can be observed in MC discharges [23]. The second deviation is an excess of the experimental ISI around 0.9–1 cycles. About 25% of the ISIs are between 0.8 and 1.2 cycles, compared to 18% for the gamma distribution. The latter result suggests that during gamma bursts, a large fraction of the MCs tend to fire at a rate close to the oscillation frequency.

The contrast between the regularity of oscillation frequency and the variability of spike discharge led us to the idea that the coupling between both activities could be dependent on neuronal frequency. This hypothesis was investigated further in the following analyses.

#### Spike phase distribution during LFP gamma oscillations depends on the MC firing rate

**Figure 2:**
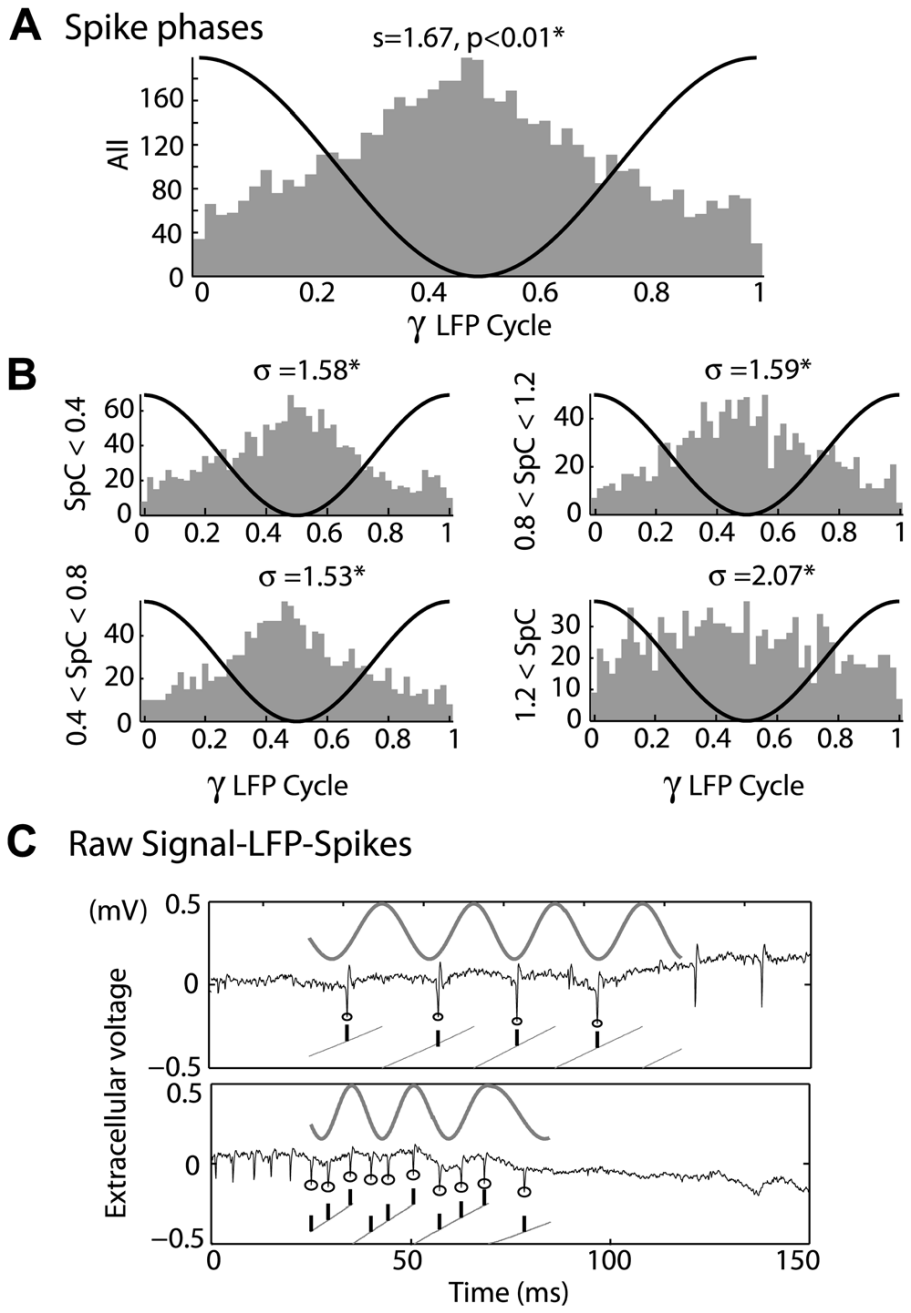
Rate dependence of spike phase distribution during gamma oscillation. A) Spike phase distribution (phase bin = 0.02 cycle) for all spikes during gamma oscillation (black line). The coupling strength was weak and statistically significant [mean = 0.472, average length, r = 0.25, circular deviation = 1.67; p<0.01, Rayleigh test)]. B) Spike phase distribution according to the mean spike rate (measured in Spike per Cycle (SpC)), (see y-label). (* indicate p<0.01 for a non-homogeneous distribution). The phase means were 0.480, 0.471, 0.486, 0.409, phase average lengths were 0.29, 0.31, 0.28, 0.11 for the respective phase distributions. C) Examples of spike trains showing regularity of spike phases at a low (upper panel) or high (lower panel) firing rate; *Black trace*, raw signal; *black circles*, spikes during gamma LFP oscillation; *grey sinusoid*, extracted LFP oscillation; *grey lines*, LFP phases from 0 to 1; *black ticks*, spike time and phase positions.

When an LFP gamma oscillation was evoked in OB slices, the distribution of GABA_A_ (Gamma Amino Butyric Acid) event times relative to the oscillation phase was phased [17] (see Fig. 4G in ref. 21). According to these observations, the MCs should receive, on average, an oscillating conductance locked to the LFP gamma oscillation. Therefore, we expected the MC firing activity to correlate with the phase of the LFP gamma oscillation. For the whole population of MCs, the spike phase distribution was peaked (Fig. 2A), with a maximum around phase 0.5 (circular deviation of sigma = 1.67 and a significantly phased distribution, P≪0.001, Rayleigh non-uniformity test). However, when the cells are segregated by their average firing rates, we observe that cells with firing rates below 1.2 SpC were better phased (Fig. 2B). We thus wondered what the difference was in the relationship between LFP fast oscillations and cells spiking at low and high firing rates.

Looking at individual spike trains on the raw signal (Fig. 2C), we noticed some particular configurations of spike-LFP phase coupling. Spike trains with ≤1 SpC (Fig. 2C, upper panel) were often stable in phase and occurred preferentially during the trough of the oscillation. On the other hand, we observed that some spike trains with >1 SpC exhibited multiple but similar phases across several oscillation cycles (Fig. 2C, lower panel). This relationship between spike phase-locking and MC firing rate raised the possibility that MC populations might be segregated according to their firing rate. We thus decided to examine systematically the relationship between phase trains and LFP oscillations while taking this observation into account.

#### Specific phase-locking and spike rate control by gamma LFP oscillation

**Figure 3:**
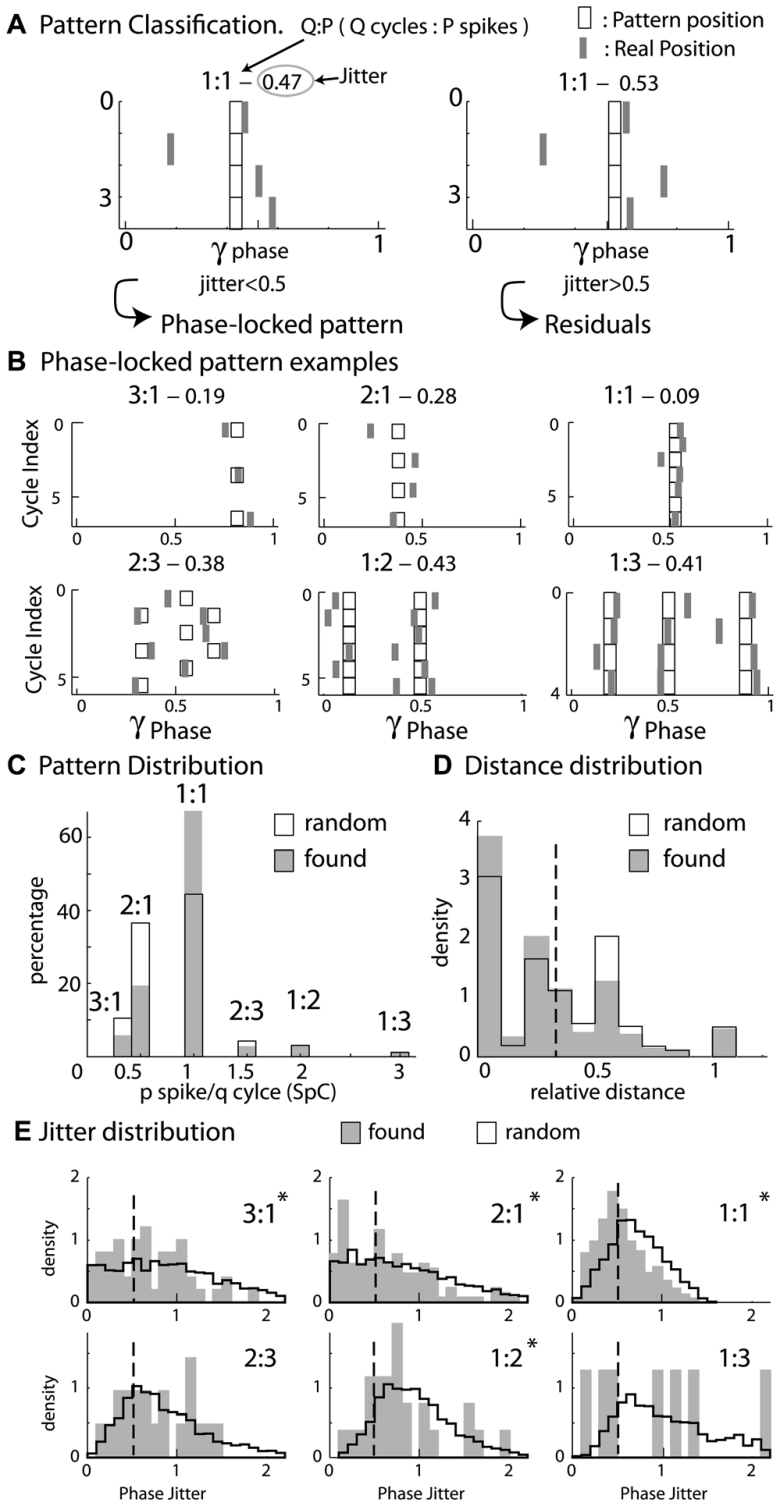
Types of phase-locking. A) Classification principles. Spike phases (x-axis) are represented by grey ticks along the successive gamma cycles (y-axis) of a LFP burst. If the spike train has a regular number of spike at each cycle (here, 1 spike at each cycle, see Methods for details), and if the phase jitter is small enough (<0.5) (left panel) in comparison to the mean phase (*black boxes*), it is classified as a phase-locked pattern (here, a 1∶1 pattern). Otherwise (right panel), the spike train is classified as residual. B) Six examples of phase-locked spike trains. Along the top of each plot is indicated the type of pattern (q∶p types–3∶1, 2∶1, 1∶1, 2∶3, 1∶2, 1∶3–where q∶p indicates p spikes during q cycles) and its phase jitter. Phase-locked patterns account for 31% of all spike trains that last during the time of at least three LFP gamma cycles. C) Distributions of experimentally found (*grey*) and randomly generated (*black contours*) phase-locked pattern types. The majority of the patterns were 1∶1 in both distributions. D) Distribution of relative distances (see Methods) from spike train phases to the closest pattern for the experimentally found (*grey*) and randomly generated (*black contours*) spike trains. *Dashed line* is the strict distance limit (0.33) under which the spike train can be considered for phase locking. Note the good agreement between experimentally found and randomly generated distance distributions. E) Distribution of phase jitter for experimentally found (grey) and randomly generated (black contours) spike trains for the respective phase-locked pattern type. The *dashed line* is the limit (0.5) under which the spike train is considered phase locked. Note that the area of experimentally found distributions under the limit was often larger than those of the randomly generated distributions, especially for the 1∶1 patterns (* indicate significant global statistical differences between both distributions, Kolmogorov Smirnov test, p<0.05).

An inspection of the spike phases (represented by grey sticks in Fig. 3A and 3B) revealed that a large number of spike trains contained spikes that had phases that were reproducible across cycles. Therefore, we automatically detected and classified the phase-locked patterns according to six q∶p types, which were 3∶1, 2∶1, 1∶1, 2∶3, 1∶2, and 1∶3, where q∶p indicates p spikes during q cycles (see Methods for details of the classification). For example in the case of the 2∶3 pattern there is a regular repetition of the number of spike at each cycle : 1,2,1,2,1,2 …, in the case of the 2∶1 pattern the repetition is 1,0,1,0,1,0… and so on for other patterns. Some examples of these phase-locked patterns are represented in Fig. 3B, which shows the periodicity of phases over a few cycles. Opposing residual trains (see methods for supplementary examples) showed a drift or at least high variability of phases across cycles. Among the 898 spike trains that spread over at least three oscillation cycles, 278 (31%) had one of the q∶p patterns. The patterns 1∶1 and 2∶1 were most frequently observed, and they accounted for 67% and 19% of the observations, respectively (Fig. 3C, grey bars).

In order to test the significance of these results, we compared our experimentally recorded patterns to patterns that were expected by chance when the phase-locked pattern detection was applied to a randomly generated spike train family with the same firing rate distribution (see Methods for details and other alternatives for random spike train generation when the ISI distribution or the phase reference are imposed). We observed that the proportion of phase-locked spike trains among all spike trains was reduced (15% in the random distribution) compared to the experimental results (31%). As detailed in the Methods section, the classification of a spike train as a pattern is a two step process. The first step of this classification relies on a “relative distance” measure. The “relative distance” between each experimental (or random) spike train and theoretical patterns was computed, which indicates how close they were in terms of spike count. The “relative distance” distribution (Fig. 3D) was similar between the experimental and randomly generated spike trains, which attests to the similarity of the spike counts of the random and experimental spike train distributions. Thus, the differences between experimental and random data must be due to the second step of the classification process, which estimates a “jitter” that measures the reproducibility of spike phases across cycles. Indeed when comparing the distribution of jitter for experimental and random spike trains, the jitter values that fell below the jitter threshold (i.e., 0.5 dashed lines in Fig. 3E) were more numerous for the experimental spike trains than those expected by chance. Additionally, experimental and random phase distributions showed significant differences for the 3∶1, 2∶1, 1∶1, and 1∶2 patterns (Kolmogorov-Smirnov test, p<0.05) (Fig. 3E). Significance was not reached for the 2∶3 and 1∶3 patterns due to the small number of experimental observations of these patterns (N = 21 and N = 8). In addition, the relative proportions of the different type of phase-locked patterns were similar (45% and 36% for the 1∶1 and 2∶1 patterns) under the experimental and random conditions. It was noticeable, however, that the proportion of the 1∶1 pattern in comparison to other patterns was significantly higher under the experimental conditions than under random conditions (Chi-square test, 70, p<0.01). Overall, these results showed that a significant population of MCs can be locked to the oscillation for various individual MC firing rates, which suggested that multiple patterns can co-exist across different cells. This finding raises the question, which we will address later, of the nature of the underlying mechanism that controls train structure in relation to the LFP.

**Figure 4:**
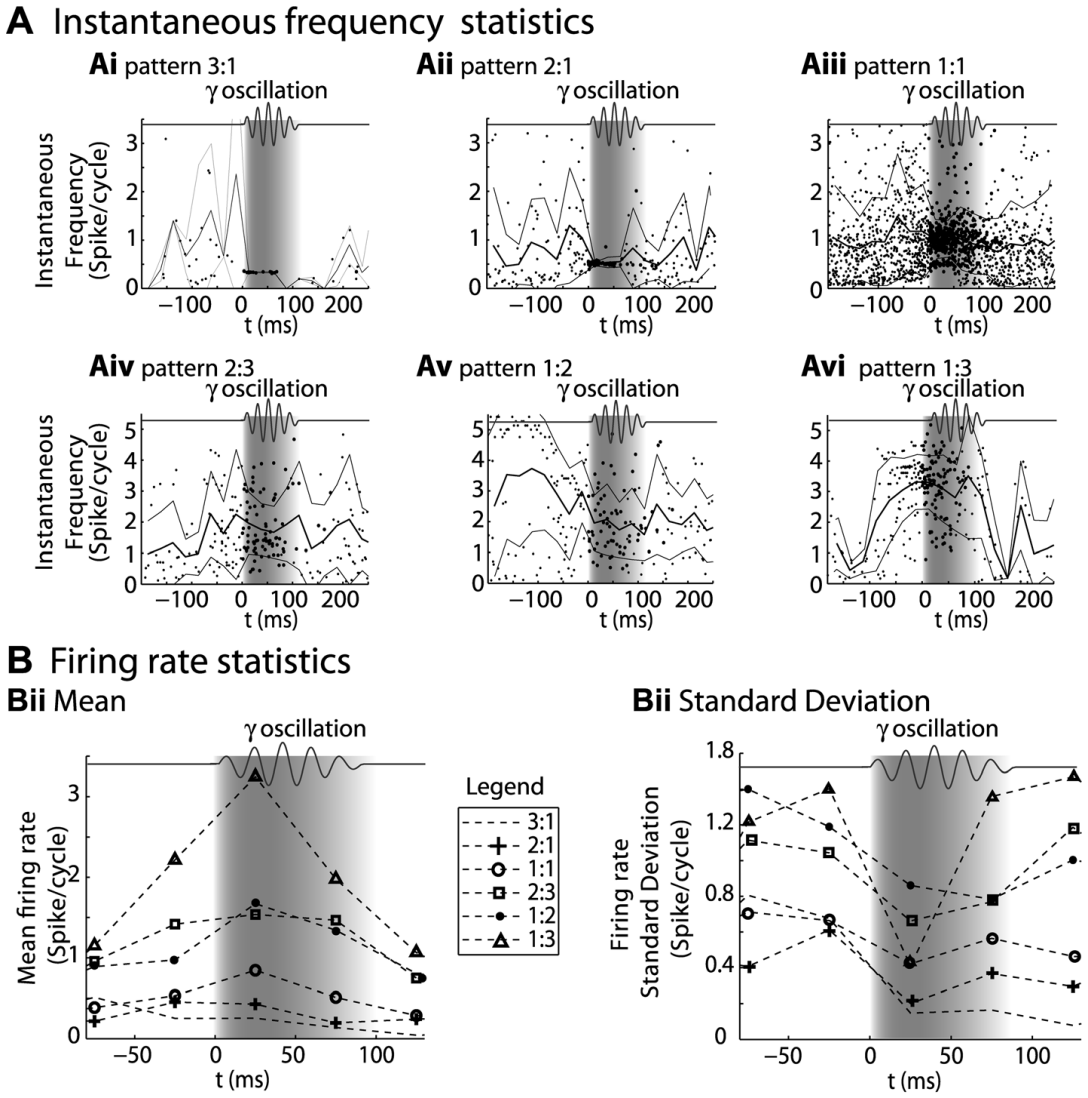
Firing rate control during gamma episodes. A) Instantaneous frequencies (1/ISI) were plotted from 200 ms before the gamma burst to 300 ms after. t = 0 at the beginning of the gamma burst, which is shown as a *grey zone* and by the symbolic oscillation atop of each graph. For each pattern type (Ai-vi), *black dots* are instantaneous frequencies (y-axes) as a function of their time position (x-axes) relative to the beginning of the LFP gamma burst. The mean instantaneous frequency (*bold line*) ± its standard deviation (*thin lines*) is estimated by averaging instantaneous frequencies over time bins of 50 ms. B) Mean firing rate averaged over 50-ms time bins (Bi) and its standard deviation (Bii) for spike trains expressing one type of phase-locked pattern (see legend for symbol-pattern correspondence). Compared to A, cells that do not fire during a bin are also taken into account in the average. Bi shows that the firing rate before and after the gamma burst was correlated with the firing rate during the gamma burst for each pattern. Bii clearly shows that the firing rate standard deviation decreases for all patterns immediately after the gamma oscillation begins.

To further characterize the relationship between cell discharge and LFP oscillation, we observed how the discharge of cells that exhibited a phase-locked pattern during a gamma oscillation evolved before, during, and after the oscillation. First, for all cells exhibiting the same type of pattern, the cell instantaneous frequency was plotted at each spike time (see Fig. 4 Ai to 4Avi for the different types of patterns, where time 0, in all cases, denotes the onset of gamma oscillations). The instantaneous frequency was measured at each spike time and was defined as the inverse of the ISI preceding the spike. The ISI unit was a cycle of LFP gamma oscillation. When the ISI fell out of the LFP gamma burst, the time reference was the mean cycle of the LFP gamma burst. This representation showed that the 3∶1, 2∶1, and 1∶1 experimental patterns (Fig. 4Ai–Aiii) were characterized by stable instantaneous frequencies during gamma episodes, whereas highly variable discharges could be observed before and after the gamma episodes. Hence, the regular discharges observed for these patterns seemed to be due to a specific process that occurred during gamma oscillations and not due to previous stability. On the other hand, the instantaneous frequency of the 2∶3, 1∶2, and 1∶3 experimental patterns (Fig. 4Aiv–Avi) exhibited a smaller decrease in variability between the “before” and “during” gamma oscillations, which suggested that the regularity detected there was more intrinsic to MC discharge and less due to the oscillation. Finally, these results were compared across the different q∶p patterns the mean (Fig. 4Bi), and the standard deviation (Fig. 4Bii) of the firing rate was estimated using a 50-ms time bin. Interestingly, phase-locked cells adopted a pattern with an average firing rate that was close to their own average firing rate before the oscillation (except for a small mismatch between the 2∶3 and 1∶2 patterns). For all types of patterns, the standard deviation of the instantaneous frequency decreased immediately after the onset of the gamma oscillation. Overall, this suggests that gamma oscillations could regularize the discharge of MCs while segregating them according to their intrinsic firing rates (i.e., cell firing rates if oscillatory input is absent). However this result does not unequivocally imply the dominance of the network oscillation on the mitral discharge, since both appeared to be intricately connected.

#### Relation between pattern types and odors

Given that pattern formation is observed during gamma bursts, we aimed to evaluate the correlation of pattern presence with odor specificity and physiological relevance. We compared the reproducibility of the pattern formation of the neuronal response during stimulation with the same odor and a different odor.

The probability of finding the same pattern in an MC response was significantly higher for responses to the same odor than for responses to different odors (probability of finding the same pattern in response to the same odor: 0.049, to different odors: 0.015, p-value<0.05, paired t-test, n = 98, see the distribution of these probability differences computed for each cell in methods). This test was performed on 98 cells among 143 cells. We selected the cells for which there were at least two recorded responses for at least two different odors. If only phase-locked patterns were considered (that is we did not take into account non-pattern spike train in opposition to the preceding analysis), then probabilities of finding the same pattern were enhanced to 0.60 and 0.49 for responses to the same odor and to different odors respectively (Kolmogorov-Smirnov test, p-value = 0.07, n = 48). Further investigations of our data at this point do not fully determine the role of locking. Nonetheless, this result reinforces the possible significance of patterns for encoding information about the nature of the odor and could help with the decoding of other structures. Although further analysis with additional data would be necessary to obtain a more quantitative understanding of the patterns' physiological function, these patterns were found to be qualitatively related to odors. The reliability (60%) of getting one pattern in response to one odor remains relative.

### Part II: modeling study

The relationships between unit activity and gamma LFP oscillations during phase-locking raise the question of whether reciprocal entrainment mechanisms exist between MC and LFP oscillations. We addressed one part of this question using a biophysical model 1) to investigate the modalities of entrainment of MC activities by granule cell activities and 2) to explain the spike train structures measured experimentally. The use of a model allowed us to perform studies that are not currently feasible with animal studies.

#### Model MCs can be differentially phase locked by an oscillation of their inhibitory input

**Figure 5:**
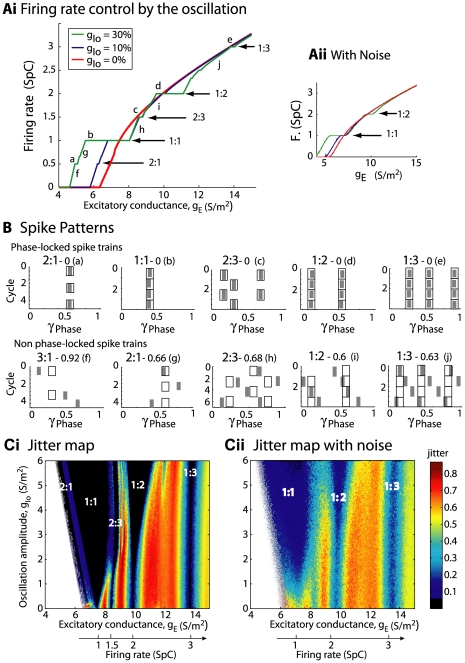
Oscillatory inhibition at 60 Hz controls MC model firing rate and phase jitter. Ai) The firing rate (y-axis) of the MC model is plotted (*red*) in spikes per cycle (SpC) for a 60 Hz oscillation as a function of the excitatory conductance g_E_ (x-axis), while the cell is submitted to a constant inhibitory conductance g_I_ = 20 S/m^2^. The firing rate was measured for two inhibitory oscillatory conductances (g_Io_): 10% (*blue*) and 30% (*green*). Plateaus appear around 0.5 SpC *(a)*, 1 SpC *(b)*, 1.5 SpC *(c)*, 2 SpC *(d)* and 3 SpC *(e)* (see arrows). Aii): Same as in Ai, but under noisy conditions. The main plateaus remained after the addition of noise. B) Examples of spike patterns along four oscillation cycles plotted using the same conventions as in Fig. 3. They correspond to different g_E_ positions along the curves drawn in Ai, i.e., without noise (see small capital letters for correspondence). C) Phase jitter map according to g_E_ (x-axis) and g_Io_ (y-axis). Like in A) g_I_ = 20 S/m^2^. Ci) Without noise, null-jitter zones (*black zones*) correspond to tongues 2∶1, 1∶1, 2∶3, 1∶2, and 1∶3 (indicated on the map). Colored zones represent the regime of non-locked spike trains with jitter >0.05 (see colored bar). Tongue width increases with g_Io_. Tongues start at g_Io_ = 0 when the unforced neuron firing rate is 1, 1.5, 2, and 3 SpC (see the firing rate-x-axis below, which corresponds to the unforced frequency at a given excitatory input). Cii) Noisy conditions. Noise tended to degrade the phase-locking, but the tongue structures persisted.

Before presenting our results, we must characterize the physiological conditions of locking. The existence of a correlation between LFP oscillations and spiking patterns presupposes neither the cause-effect relationship between the two activities nor the biophysical mechanisms of interactions in the bulbar network to explain the phase-locked patterns. In vitro experimental results by Lagier *et al.* (2004) [17] (see their Fig. 4G) showed that, during LFP gamma oscillation, MCs can receive an average level of inhibition due to tonic activation of GABA_A_ receptors, along with a superimposed phasic inhibition that occurs at gamma frequency. From the synaptic connectivity and the individual synaptic strength, we were able to estimate the average level of tonic inhibitory conductance (called g_I_) to be on the order of 20 S/m^2^ and the amplitude of the oscillation (i.e., the phasic inhibitory conductance, called g_Io_) to be up to 30% of the average level (see Methods for details about the MC model and all of the parameter estimations). We tested how the response to sensory inputs, which was mimicked by a constant and tonic excitatory conductance (called g_E_), and was influenced by the inhibitory sinusoidal conductance (g_I_+g_Io_) oscillating at a frequency (f_osc_) in the gamma range (Fig. 5A). This inhibitory conductance oscillating at 60 Hz was set to 0%, 10%, or 30% of the average inhibitory conductance g_I_ (i.e., g_Io_ = 0, 2, or 6 S/m^2^, respectively). As shown by the different plateaus on the curves of the cell response (Fig. 5Ai), the occurrence of such an inhibitory conductance stabilized the firing rate. The frequencies at which the firing rate was stabilized corresponded to the q∶p patterns that occurred around frequencies given by p/q multiplied by the frequency of the imposed oscillation (60 Hz). Throughout this analysis, q and p are distinct integers chosen among {1,2,3}. These frequencies corresponded to frequencies in the experimental phase-locked patterns. The larger the oscillation amplitude, the wider the stabilized plateau (see the curves from blue to green in Fig. 5Ai). Interestingly, the inhibitory oscillation was able to increase the firing rate (the plateau for 1∶1 lies above the curve with g_Io_ = 0) and could evoke firing in a silent cell (Fig. 5Ai). Under noisy conditions (Fig. 5Aii), the conductance-frequency response was partially preserved, but the smallest plateaus (for 2∶1, 2∶3, and 1∶3) disappeared. Figure 5Bi shows, in the noiseless case, phase diagrams of trains that were extracted from each of the plateaus mentioned in Fig. 5Ai (referred as “a”–“e”). The relatively good agreement with experimental observations [e.g., compare the experimental spike trains in Fig. 3B with the simulated trains in Fig. 5B] suggests that MCs can be driven effectively by an oscillatory input during gamma oscillations. Additional spike trains that were extracted from the non-plateau portions of the firing rate response curve (referred as “f”–“j” in Fig. 5Ai) are shown in f through j of Fig. 5B. These results indicate that MCs are entrained by an oscillatory inhibitory conductance mainly when the neuron fires at the same frequency as the imposed 60 Hz oscillation, and that this entrainment can also explain the patterns that are observed in our experiments. This result is partly specific to the model, since other intrinsic properties for other neurons would not lead to a 1∶1 entrainment. Indeed, all cochlear neurons have dissimilar entrainment properties with regard to the frequency of entrainment [25].

We next analyzed the influence of the oscillation amplitude on spike phase-locking. For this purpose, a color map was used to plot the phase jitter, which was defined in the same way as it is used to classify spike trains as a phase-locked pattern or not, as a function of the amplitude of the oscillating inhibitory conductance, from 0 to 30% of the average of the inhibitory conductance (y-axis) and the amplitude of the MC excitatory input conductance (Fig. 5Ci). The so-called “Arnold tongue” for 1∶1 [20] (zero jitter, which would occur in perfectly locked spikes, in black and noted directly on graph) was the widest, while the tongue for 1∶2 was the second widest. The higher the oscillation amplitude, the wider the tongues for 1∶1 and 1∶2. These tongues were partly preserved under noisy conditions (Fig. 5Cii), but tended to shrink and disappear at low levels of oscillatory inhibition. As a consequence, as the oscillation amplitude tends to increase, a higher proportion of cells may be entrained by the oscillation, even if the cells' intrinsic firing rate is far from the frequency that is imposed by the entrainment (see the doubled x-axis, below). Therefore, a larger MC population can be recruited when the oscillation amplitude increases.

**Figure 6:**
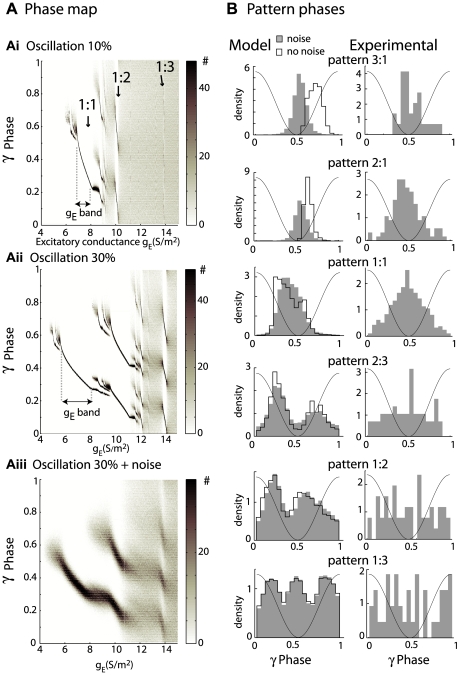
Model phase distribution and comparison with experimental phases in phase locked patterns. Ai) Model spike phase (y-axis) distribution is plotted like a phase map (see grey scale bar) as a function of g_E_ (x-axis). For a 10% oscillatory inhibition (g_Io_ = 2 S/m^2^, g_I_ = 20 S/m^2^), the MC model exhibited clear preferential phases (*dark spots*; arrows indicate tongue q∶p). Aii) Increasing the inhibitory amplitude to 30% (g_Io_ = 6 S/m^2^) value for g_Io_ led to an increased g_E_ band (see the horizontal double arrow), for which the phases were locked. Aiii) Noisy conditions, which were the same levels of inhibition as in (Aii), caused the phases to scatter around the tongue phases. B) Comparison of model (*left panels*) and experimental (*right panels*) phase distribution for spike trains classified as phase locked patterns (one row for each type of pattern). The pattern spike phases are extracted from Fig. 5Ci for the noise free conditions (*black contour*) and from Fig. 5Cii for the noisy conditions (*grey bars*) under the synaptic conditions g_Io_<4 S/m^2^, g_E_<15 S/m^2^ and g_I_ = 20 S/m^2^.

The next step was to understand the origins of the inter- and intra-pattern phase variability. For this purpose, we represented the spike phase variations according to the driving excitatory conductance g_E_ for two levels of inhibitory conductance, namely 10% (Fig. 6Ai) and 30% (Fig. 6Aii). Under these noiseless conditions, the 1∶1 tongue (arrows) had phases that varied continuously from 0.5 to 0.2, as the excitatory conductance increased. We noticed that the range of possible phases for each tongue was not greatly affected by the oscillation amplitude, whereas the conductance range (the g_E_ band indicated) over which the MC was entrained increased with the oscillation amplitude (compare Fig. 6Aii to 6Ai). Phase-locking was robust to noise, and the phase distribution persisted, although less sharply, for high levels of noise (Fig. 6Aiii). The model supported the experimental observation that MCs can be phase-locked at various frequencies and under noisy conditions. Finally, comparing the model prediction to our experimental results at the population level is impossible because of the lack of data on the distribution of the input excitatory conductance g_E_ in the MC population. Thus, in Fig. 6B we present only side-by-side comparisons of those spike phases that can be predicted by the model (left column) for each type of pattern and their experimental counterparts (right column). The phases of the spikes of the model come from the patterns that are detected under quiet conditions (contour) (corresponding to Fig. 5Ci) and noisy conditions (grey) (corresponding to Fig. 5Cii). For a better fit, we used only the patterns evoked when g_Io_<4 S/m^2^). We observed agreement between theoretical and experimental data especially under noisy conditions. Indeed, whereas without noise in the model, some differences are observed between the mean phase predicted by the model for the 3∶1, 2∶1 and 1∶1 patterns and those observed in the data, introducing some noise tends to decrease these differences in the mean phase between the model and the experiments. The spike phases of patterns with high firing rates, that is, 2∶3, 1∶2 and 1∶3, seem less sensitive to noise. In addition, the difference of the phase distribution width between the model and experiments could be explained by the measure variability of the gamma phase that was recorded from different depths and that will be further discussed later.

#### Synaptic input parameters governing the entrainment

Having shown that our model can reproduce our experimental results well, we further used it to explore how MC entrainment should depend on network parameters or MC intrinsic parameters.

**Figure 7:**
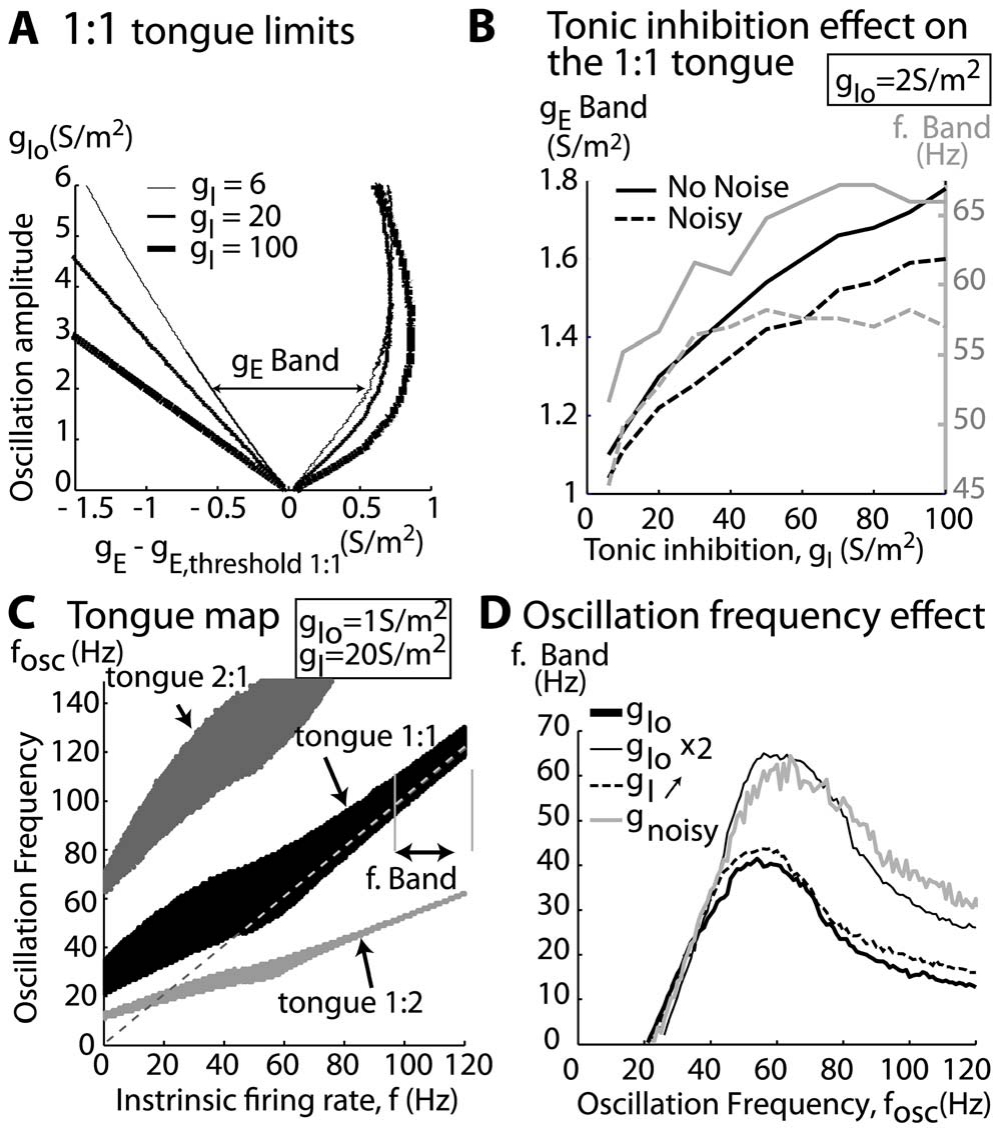
Influence of synaptic input parameters on MC model locking. A) Tongue 1∶1 limits. The edges of tongue 1∶1 are shown for various levels of global inhibition g_I_ (6, 20, 100 S/m^2^) in the absence of noise. Values of g_E_ corresponding to tongue edges are shown relative to g_E,threshold 1∶1_, which corresponds to the level of excitatory conductance g_E_ necessary to induce a neuronal intrinsic firing rate equal to the oscillation frequency (that is when g_Io_ = 0). This level varies with g_I_ that is why the reference is needed to study the effect of various levels of g_I_. B) Tonic inhibition effect. Representation of tongue 1∶1 width for g_Io_ = 2 S/m^2^. The g_E_-band (*full black* line in conductance units, left y-axis) and the f-band (*full grey line* in frequency units, right y-axis) are shown as a function of the global inhibition level g_I_. The f-band is the range of unforced frequency that can be locked by the oscillation. Dashed lines correspond to noisy conditions and g_Io_ = 1 S/m^2^. C) Tongue map. Phase-locked zones are shown for the tongues 2∶1 (*dark* grey), 1∶1 (*black*), and 1∶2 (*light grey*) in the plane that is represented by the intrinsic MC frequency, which is determined when g_Io_ = 0 (x-axis) and the frequency of the oscillation (f_osc_) (y-axis). For these results, g_I_ = 20 S/m^2^, and g_Io_ = 1 S/m^2^. The *dashed line* is y = x. D) Oscillation frequency preference for locking. The 1∶1 tongue f-band width [indicated by arrows in (C)] is plotted as a function of f_osc_ using a *bold black-line* with the parameters in (C). Various conditions are tested, such as the amplitude of g_Io_, g_I_ and g_Is_ (see details in the figure). MC entrainment was always maximal between 50 and 70 Hz for any value of g_I_, g_Io_, and noise.

First, we studied the influence of the excitatory-inhibitory balance. The level of inhibition received by an MC is not known, and it is difficult to infer using standard experimental approaches (see estimation in Methods). It may vary within a range depending on the level of recurrent, lateral, and centrifugal inhibition that are present in the OB network, and these networks are highly variable and modulated in their nature and function. To explore the consequences of the average inhibition for the entrainment, under noiseless conditions, we set the level of background inhibition to different values (g_I_ = 6, 20, 100 S/m^2^). This range of values corresponds to the range of influence, ranging from a small effect to a complete shunting effect on the spiking activity. We then measured the entrainment for the 1∶1 tongue by representing the entrainment diagrams as a function of MC excitatory input and the inhibitory oscillation amplitude (see Fig. 7A). The 1∶1 tongue grew wider as the average level of inhibition increased. The tongue width was measured by the g_E_ band (Fig. 7A), and computed with an oscillation amplitude of 2 S/m^2^ that increased with the average inhibition level (Fig. 7B, full black line, left y-axis). Alternatively, a given set of conductances (g_E,_g_I_) corresponds to a given MC firing rate, and thus the g_E_ band can be converted into a f-band that describes the range of firing rates (in the absence of oscillation) at which the MC are entrained by the inhibitory oscillation in a 1∶1 pattern. Again, the width of this f-band increases with the average inhibition level (Fig. 7B, full grey line, right y-axis). These results were robust under noisy conditions (see dashed lines in Fig. 7B). Taken together, these findings reveal that a higher level of background inhibition of MCs makes them more capable of being entrained.

Second, we showed that an oscillation frequency in the gamma range is optimal for entrainment. Experimental data showed frequency stability in the LFP oscillation around 60.7 Hz (Fig. 1D). Since the frequency of gamma oscillations varied little under our experimental conditions, as well as under other conditions [10],[18], we wondered why the frequencies had these particular values. We tested how the model reacted to other imposed frequencies (f_osc_) of the inhibitory conductance ranging from 5 to 120 Hz and then analyzed the zones for which the MC model was entrained, according to both its intrinsic firing rate and the frequency of the oscillation. Such zones are represented for the 2∶1, 1∶1, and 1∶2 tongues in Fig. 7C. These results demonstrate that the MC models can be entrained by any frequency oscillation in a 1∶1 pattern, as long as the oscillation frequency is close to the intrinsic firing rate of the MC. This relationship is reflected by the fact that the 1∶1 tongue covers the diagonal f_osc_ = f_intrinsic_ in the graph in Fig. 7C. More noticeably in the case of the 1∶1 tongue, the bandwidth (f-band) was largest when f_osc_ was in the 40–60 Hz range. In contrast, for the 2∶1 and 1∶2 tongues, the maximal entrainment was observed for oscillation frequencies around 120 Hz and 30 Hz, respectively, which correspond to an MC intrinsic firing rate of about 60 Hz in both cases.

We further explored the broader range of entrainment that was observed when the MC intrinsic activity was close to 60 Hz. Due to the importance of the 1∶1 pattern in both the experimental and simulated results, we focused on the frequency band (f-band) of the 1∶1 tongue. The bandwidth is represented as a function of the oscillation frequency in Fig. 7 (D, black thin line). When the amplitude of the oscillation was varied from 5% to 10% of the global inhibition level, the peak at around 60 Hz persisted. Varying the average inhibition level (g_I_) or adding some noise to the system did not change the position of the peak around 60 Hz. These results indicate that MCs may be preferentially entrained by oscillations at 60 Hz, and that they are robust to variations in the parameters for synaptic stimulation of MCs.

**Figure 8:**
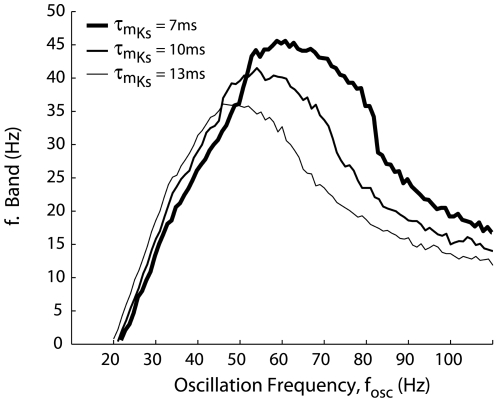
MC-intrinsic properties control the range of maximal entrainment. The width of the intrinsic firing rate range for which the MC model is locked (i.e., the f-band width) is plotted as a function of f_osc_ (as in Fig. 7D) for different values of the slow K channel activation time constant. τ_mKs_ = 10 ms was the default in previous figures, τ_mKs_ = 7 ms (*thin line*) indicates a peak drift to faster frequencies, and τ_mKs_ = 13 ms (*bold line*) shows peak drift toward lower frequencies.

The last step was to explore how the intrinsic properties of the MC model were potentially responsible for the 60 Hz preference. For that purpose, we varied the time constant of the slow potassium channel, which was responsible of the MC model subthreshold resonant properties, from 7 ms to 13 ms. We observed that the peak of maximal entrainment could drift from lower to higher frequencies when this time constant was decreased from 13 to 7 ms (Fig. 8). As a comparison, there is not such a peak of maximal entrainment with an integrate and fire model; the profile of the curve there is only decreasing. We concluded that the intrinsic properties of the MC model may be responsible for the gamma-range frequency preference for entrainment. Although it is not currently possible to alter the intrinsic properties of the neuron experimentally, the model that we used in this study demonstrates that the intrinsic properties of the MCs can interact with network properties, thereby leading to specific entrainment of MCs, the consequences of which will be discussed in the Discussion section.

## Discussion

We found that specific MCs produced spike trains that were locked to the gamma LFP oscillations in specific pattern types. Such phase-locked cells exhibited particular firing rates that were stabilized by the occurrence of gamma oscillation. In most cases, the firing rate was one spike per cycle (SpC).

Using a MC model with in vivo-like conditions that receives a small inhibitory 60 Hz sinusoidal conductance, we showed that these observations were characteristic of an entrainment process. The model exhibited similar phase-locked patterns of entrainment, with maximal entrainment for one SpC patterns. The spike phase distributions were similar in the model and the experimental data, and corresponded to the minimum inhibitory conductance associated with the LFP oscillation. This model suggests that there is an efficient process of entrainment in the OB that can make MC activity coherent across various OB areas during gamma oscillations. The relevance of such a mechanism is in the possibility of using it to ensure an underlying basis for neuronal assembly formation that can be used more generally by the nervous system [26].

The use of a robust biophysical model allowed us to predict the optimal conditions for an entrainment occurrence if

1. the chemical features of the odor belong to the chemoreceptive field of an MC, such that its excitatory response will be strong enough to elicit spiking;
2. strong tonic network inhibitory interactions that are intrinsic to the bulb and/or generated by the cortex feedback are generated, such that they will balance this excitation and will, therefore, provide what is called a level of background inhibition; and
3. the distribution of the IPSCs that are received by the MC is sufficiently well phased, which leads to a sufficiently large oscillating conductance, then a single MC has a good probability to be entrained and locked into the oscillation. As a result, its spike phases become tightly controlled by the oscillation.

### Contribution of our work to existing experimental data

Our work provides converging arguments in favor of a MC entrainment, suggesting that they receive an oscillatory input in vivo. Although several groups have focused on the temporal relationship between spikes and LFP, the exploration of this entrainment phenomenon has only been partly addressed. The analysis of Eeckman and Freeman [16] differed due to the multi-unit nature of their signal. Kashiwadani et al. [18] focused more on a coding hypothesis rather than a dynamic hypothesis and used an artificial sniff, which likely affected the bulbar dynamics. One in vitro study has explored the spike/LFP relationship [17]. The network connectivity and dynamic conditions, however, could be very different from those in vivo, and it is difficult to compare in vitro oscillations to those under in vivo conditions.

One potential criticism of our work is the usefulness of such a mechanism under some behavioral conditions, such as where odor presentation induces a decrease in gamma oscillations [14]. The fact that the amplitude of gamma oscillations decreases under particular conditions does not indicate that gamma oscillations are unnecessary for the system. Indeed, the amplitude decreases under particular conditions; however, beta oscillations are simultaneously enhanced, which suggests that the olfactory bulb and cortex together work in a different manner [27],[28]. There are other behavioral conditions in which gamma oscillations do not decrease but rather increase with odor presentation [10]. Experimental conditions vary widely depending on the employed paradigm, which can induce different sniffing strategies [29] or different neuromodulatory control of the MC/GC loop [30]. These different conditions are certainly responsible for the variation in the expression of gamma and beta oscillations. The aim of our study was to explore how spikes and oscillation can be phase-locked in the more general situations in which gamma oscillations are present. It would be an interesting second step to perform the same study for beta oscillations and to look for another mechanism that could be responsible for the observed spike/beta oscillation synchronization [31].

### Basis of the model and neuronal mechanisms

Our MC model was fitted to reproduce the quantitative and dynamic properties of MCs, based upon recent findings on MC membrane activity. It is consistent with experimental data [17], [32]–[34] and includes essential features of existing models [35]–[37]. In our model, entrainment was produced by a relatively weak oscillatory inhibitory conductance. When the cell has an input well above the threshold, it fires regularly and even periodically in response to a constant input. Under these conditions, and when the oscillatory input is sufficiently weak, the neuronal response can be predicted from the phase response curve (PRC) [38],[39], which can also predict some characteristics of the tongues [40]. Since the PRC of our MC model agrees with the experimental one [41], it is likely that our model can accurately predict the MC response to such synaptic inputs. The synaptic mechanisms hypothesized in our model are based on observations made by Lagier et al. (2004) [17], who reported that a fraction of the IPSPs received by the MCs were phase-locked relative to gamma oscillations. Other possible mechanisms that could affect the MC membrane potential during gamma oscillations have been proposed: (1) a synchronous inhibition resulting from GABA release from astrocytes [42], (2) a synchronous excitation of the MCs by glutamate spillover onto lateral dendrites [43],[44], and (3) a direct influence of the LFP on membrane voltage [45],[46]. Of these possible explanations, the functional and dynamic relevance of the interactions between MCs and GCs [47] are the most likely processes to occur during gamma oscillations.

Aside from this observation, an important characteristic of our model is that the time constant of the single inhibitory inputs does not play a role because it is the frequency of the oscillatory modulation of the whole inhibitory input that imposes the locking frequency.

It is important to insert this mechanism into a more realistic model in order to take into consideration the spatial distribution of the physiological mechanism in dendrites. The presence of high frequency bursts of spikes in MCs is probably not due solely to the excitatory drive from the sensory input, but also to additional conductance levels beyond those present in the model. For example, the activation of an intrinsic conductance, such as T channels, would facilitate the emergence of high frequency bursts like those in our recordings.

### Explanation of model predictions and experimental recordings

Neuronal phase-locking and pattern formation have already been described both theoretically [20],[21],[48] and experimentally [26],[49]. Our study, performed in the context of the OB with a resonant neuron, however, sheds light on important aspects that are specific to this system.

First, the simulation results provide some precise explanations regarding electrophysiological properties that are not directly accessible by experimentation. The phase of the MC spikes relative to LFP oscillations is precisely distributed in the ¼ to ½ part of the LFP cycle due to their level of synaptic drive. The phase distributions extracted from simulated data (Fig. 6A) are more precise and go beyond the data that was obtained in the OB [14],[17],[18], as well as our own experimental results. We wish to point out, however, that our experimental measurement of gamma phase was not totally reliable from one particular depth to another, and could have up to ¼ of a cycle of phase shift. Indeed, gamma oscillations are generated by membrane currents flowing on either side of the MC layer, and the simultaneous recording of a gamma oscillation constantly shows a ¼ cycle phase shift when it is recorded up to 100 µm from the MC layer (Fourcaud-Trocmé and Buonviso, personal communication). In our recordings, we could not control the exact position of each electrode relative to the MC layer. As a consequence, these recording constraints, as well as the noise level, are likely to be the source of the larger spread of phases during the phase-locked experimental pattern when compared to the model prediction (see Fig. 6B). This can also explain the small discrepancies with recordings from other studies in which the LFP recording position relative to the mitral cell layer was different or more precisely controlled [16]–[18]. In all cases, however, our results are still qualitatively comparable.

Second, lower levels of excitatory conductance in the model were predicted to induce a slight phase lag (corresponding to ∼5 ms) between the neurons, as shown in Fig. 6A. This phase difference is observed in the cross-correlograms that were presented by Kashiwadani et al. (1999) [18] (their Fig. 5A), and it may be achieved by differential activation of the glomerulus [18],[50],[51]. Our predictions are able to explain the result of this previous study.

The model predicts that phase-locked or residual spike trains may depend on the degree of detuning (i.e., the difference between the neuronal intrinsic firing rate and the external network frequency), the strength of coupling, and the level of noise. The continuity between the locked and residual spike trains suggests that entrainment of MCs may be fine-tuned during the gamma oscillation, and may corroborate the locking that was observed in the fish OB [13]. However, only simple phase-locking has been found in these species.

Finally, in our model, increasing the global level of excitatory-inhibitory conductance favors entrainment (see Fig. 7A–B). Under anesthetized conditions, the maximal conductance should be directly related to the maximal activity of the peripheral excitation [51],[52] and to the maximum of synaptic inhibition [53], which corresponds to the inspiration/expiration transition. Taken together, these data imply that entrainment should be the strongest at this transition, which is what was observed with the gamma wave appearing around this point (Fig. 1). Interestingly, odors that elicit low theta activity (respiratory modulation), which potentially reflects weak OB activation (e.g., low-concentration odors), generally failed to elicit gamma oscillation [9], suggesting that the theta oscillation could gate gamma activity.

### Implications on the origin of the gamma oscillation

In the OB, MCs are directly interconnected only within glomerulus [54]–[57] and not across bulbar areas. Instead, MC spikes induce a depolarization of some GC spines, which propagates through parts of the cell to other spines and triggers both synchronous [58]–[62] and asynchronous [36], [55], [63]–[65] GABA release from granule dendrodendritic synapses onto other MCs. This peak of GABA release is about ¾ of the gamma cycle (∼ = 12 ms) after the peak of spiking (according to the spike (Fig. 4E) and IPSP (Fig. 4G) phase distributions in [17]). The slow component of inhibition [55], [64]–[66] may contribute to the global tonic inhibition level that is present in the OB. This synaptic activity suggests that the effective interaction between MCs is inhibition, and that the OB could be approximately modeled as a network of inhibitory coupled MCs.

Our results raise the question of the origin of gamma oscillatory activity in the OB. Do entrained MCs participate in the creation of this rhythm? If so, how? Two models have been recently proposed [35],[36]. In [35], a network of inhibitory coupled MCs is applied to in vitro recordings, in which the gamma oscillation is continuous and evoked by single stimulation. In [36], the particular tendency of MCs to synchronize their firing when their stochastic inputs become correlated was studied. Neither of these models, however, has been shown to induce the spiking activity that we report here. This is also the case for the general mechanisms that have been proposed for oscillation generation in inhibitory [67]–[69] and excitatory-inhibitory networks [69]–[71]. It has to be noted that, in these studies, a large heterogeneity in the population intrinsic firing rates usually prevents the formation of an oscillation, and that the case of a resonant neuron is not considered.

Our results, however, suggest the following two hypotheses. First, if MCs behave like oscillators, a possible mechanism for the emergence of the oscillation may be provided by the transition towards synchrony, a phenomenon that is generally observed in a network of coupled heterogeneous oscillators [20],[72]. Second, the model predicts that intrinsic MC properties (according to Fig. 7D and 8) lead to a maximal entrainment in the gamma frequency range. Indeed, a non resonant neuron (i.e. an integrate-and-fire model) does not exhibit this behavior (data not shown). From this property, it can be seen that among the MC population that exhibits quite heterogeneous frequencies (see Fig. 4), MCs firing with intrinsic firing rate in the range of 40–70 Hz are better entrained than MCs firing at lower or higher frequencies. The rhythm could be created when this entrained MC population becomes sufficiently large.

### Coding implications

Other studies in anesthetized rats have shown that MCs can be segregated into two populations according to whether they phase-lock to gamma oscillations or to beta oscillations, which is likely due to their position relative to the receptive field of the odor [31]. Here, we show that, among MCs that lock to the gamma oscillation, locking is finely tuned depending on the various conditions that control MC entrainment, one of them being the nature of the odor. A subset of the MC population can therefore be entrained and phase-locked at gamma frequencies with patterns depending on the intrinsic firing rate of the MC, which is similar to the previously described rate-specific synchrony [26]. The resulting phase-locked activity map may overlap with the glomerular activity map. This is suggested by our results, which show that pattern types are partially reproducible for the same cell and the same odor. The glomerular map is likely forwarded to MCs as a “rate code” map that is readable by downstream structures. The phase-locked activity map, however, appears to be more elaborate than the simple glomerular map. In particular, the main characteristic of the phased-locked activity map is that it can be modulated by various parameters: such as the conductance level (Fig. 7), the MC intrinsic firing rate (Figs. 4 and 5), and/or the degree of GC-MC coupling (Fig. 5C). It is therefore likely that this phase-locked activity map is modifiable by plasticity mechanisms and central control [10]. Based on the adjustment of the spike phases, the mechanisms described here would result in population activity that is readable by decoding structures which function as coincidence detectors. Pyramidal cells of the piriform cortex have such detection properties [73]. In addition, synaptic integration by pyramidal dendrites is more sensitive to pattern-like inputs, as shown in other sensory systems [74],[75]. Thus, our model of a phase-locked map of MC activity across the OB supports the idea developed by Zou and Buck [76] that pyramidal cells could integrate odorant features using a combination of coincident MC inputs (as argued also in [77]) in a way similar to the mushroom bodies of insects [78],[79].

## Methods

### Ethics Statement

All experiments were performed in accordance with the guidelines of the European Communities Council.

### Preparation and recording

Male Wistar rats (150–350 g) obtained from Charles River Labs (L'Arbresle) were anesthetized with urethane (i.p. 1.5 mg/kg, with additional supplements as needed) and placed in a stereotaxic apparatus. The dorsal region of the OB was exposed. Bulbar activity was recorded as a broadband signal (0.1–5 kHz) using 16-channel silicon probes (NeuroNexus Technologies, Ann Arbor, MI) with a homemade, 16-channel DC amplifier. The data were digitally sampled at 10 kHz and acquired on a PC using the IOtech acquisition system (Wavebook, IOtech, Cleveland, OH). Probes were placed in the lateral or medial part of the OB at a depth that maximized the number of channels located in the mitral cell layer (MCL). The respiration signal was recorded using a homemade flowmeter based on a fast response time thermodilution airflow sensor. Odors were delivered through a dilution olfactometer (440 ml/min). The recording protocol was as follows: 5 s of spontaneous activity, 5 s of odor-evoked activity, and 5 s of post-stimulus activity. Each sampling included stimulation by simple linear aliphatic compounds. The varying features of odorants were either the number of carbons in the main chain (chain length: 5, 6, 7 and 10 C) or the functional group associated with the chain (alcohol, ester, aldehyde or ketone). Additional odors have been used, including isoamyl acetate, p-cymene, and eugenol. All odors were delivered in front of the animal's nose at a fraction of 18.10^−2^ of the saturated vapor pressure. The time delay between each odor presentation was at least one minute.

### Data processing

#### Respiratory signal

The signal was processed as previously described [80]. Briefly, respiratory cycles were extracted, and, for each one, the time was converted into a phase that had a value that varied as [−π, 0] for inspiration and as [0, π] for expiration. The transition point between inspiration and expiration (I/E) corresponded to the 0 phase, which was detected as the point where the signal crossed the 0 value and which corresponded to the point where there was no variation in pressure.

#### LFPs

LFPs were obtained by band-passing the recorded signal at 5–200 Hz. To preserve both time and frequency information, we used a time-frequency representation (TFR) that was based upon continuous wavelet transformations, as previously described [81]. Briefly, the LFP signal was convoluted by complex Morlet's wavelets [82] with a time resolution of 5 ms and a frequency resolution of 1 Hz. Using a wavelet ridge extraction method coupled with a high time-resolution TFR, each gamma oscillatory epoch of the LFP was extracted using an energy threshold to detect its beginning and end. This procedure allowed a reasonable estimation of the phasic, temporal, and frequency features of these oscillations. The TFRs were normalized to the energy measured during the spontaneous activity in the 5 s before odor presentation.

#### Spikes

Signals from individual electrodes were amplified (gain 1,000×) and filtered from 300 to 5000 Hz. Multiunit activity consisted of a few neurons on each electrode. Spikes were clustered off-line using a semi-manual routine. We chose to use only the units that showed a signal/noise ratio ≥5 and to sort cells according to their spike amplitude. Consequently, the number of units that were retained for analysis was restricted to 1–3 per electrode. This conservative procedure resulted in the analysis of a relatively limited number of units but provided high quality data. Additional details of the method are given in Cenier et al. [31]. Moreover, autocorrelograms of spike trains that belonged to presumed single units confirmed that a refractory period exists and precluded the possibility to have multiunit recordings included in our single unit ones. The ISI histogram in Fig. 1E shows this refractory period at the population level.

#### Spike phases

To study relationships between spike and LFP, (these were always recorded from the same electrode), we converted spike times into phases that were computed in cycle units (i.e., the phase increased by one at each cycle) during each LFP gamma oscillation epoch. This change in the time axis allowed the interspike interval (ISI) (respective firing rate) to be measured in cycle units (respective spike per cycle (SpC)). Using phases instead of time allowed us to discard temporal or frequency irregularities across oscillatory epochs.

### Statistics

#### Phases

For a given ensemble of spikes (*N*) with relative phases 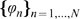, the phase distribution was represented as a histogram (bin size: 0.02 cycle). To analyze this distribution, we used circular statistics [83]. The mean phase 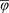 is defined by:
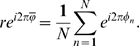
(1)The circular standard deviation uses the modulus (r) of this sum and is given by 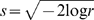. The value of *r* increases from 0 to 1 as the phase distribution changes from being uniform to sharply peaked.

A Rayleigh uniformity test was used to calculate the probability that the data were uniformly distributed (null hypothesis).

Using, 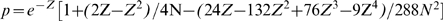, with 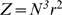, the level of significance for non-uniformity was set at a p-value<0.001 [83].

#### Interspike Intervals

The interspike interval (ISI) distribution was compared to the density function of a random variable process following a gamma distribution that was reconstructed in order to have the same mean and a random refractory period (with a Gaussian distribution that had a mean of 3 ms and the standard deviation at 1 ms), such that the refractory period is sorted each time the sorted ISI falls below the refractory period [84].

In addition, we considered both the instantaneous frequency, which was defined for each spike time as the inverse of the preceding ISI, and the mean firing rate, which was given by the average number of spikes that fell within a time bin of 50 ms. The ISI unit was a cycle of LFP gamma oscillation. When the ISI fell out of the LFP gamma burst, the time reference was the mean cycle of the LFP gamma burst.

### Detection and classification of phase patterns

#### Summary of pattern definition

**Figure 9:**
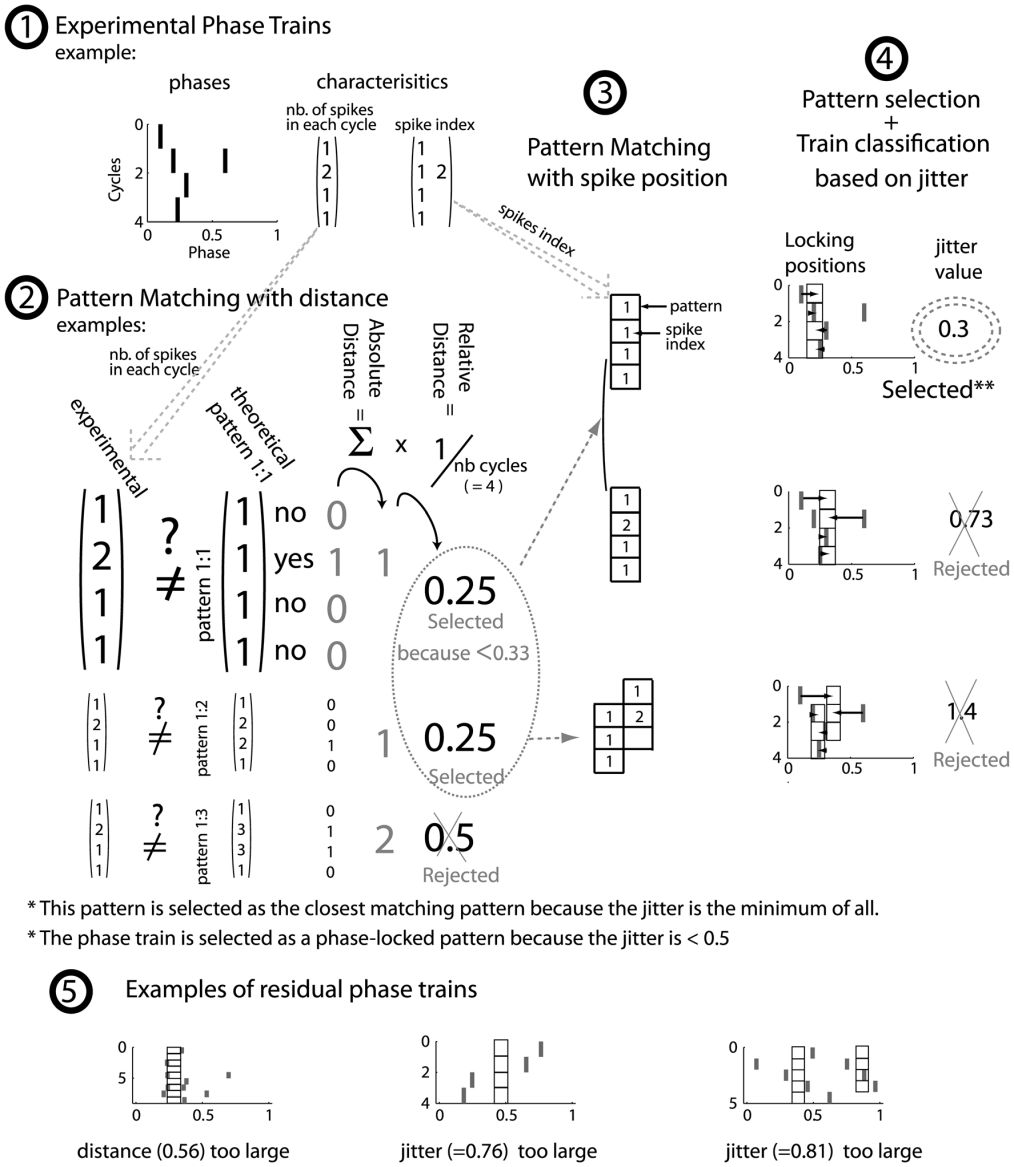
Method for detection of phase-locked patterns. Step by step description using an example: (1) A spike train taken from experimental recordings is represented with its phases (x-axis, top left) along the successive gamma cycles (y-axis). This train was characterized by the number of spikes in each cycle (left matrix), and we allocated different indexes for spikes in the same cycle in the order in which they appeared in the cycle (right matrix). (2) Independently, a pattern sequence generator created all phase trains that have the same cycle length as the experimental one. In this case, the length was four. Three examples of all these generated pattern sequences are shown as column vectors (theoretical pattern column), each of which is affiliated with a particular pattern (e.g. 1∶1, 1∶2, 1∶3). The absolute distance between experimental spike train and theoretical pattern was measured by summing the result of the equality test over all of the cycles of the train. In terms of spike number, if the spike numbers are identical, then the test result is 0, otherwise it is 1. The relative distance was obtained by dividing the absolute distance by the number of cycles that were covered by the train. At this step, we eliminated the pattern sequences with a relative distance > = 0.33 (e.g. the last line pattern 1∶3 example). (3) All of the position possibilities were then considered for the spike (index) position of the experimental train in the selected patterns (grid). Single or multiple correspondences are shown for each selected pattern. (4) Based on these positions in the hypothesized pattern, we empirically estimated the mean phase of the pattern from spike positions. For example, in the first plot (top right), the black boxes are the mean phase of spike index 1. A jitter was calculated based on the distance of each spike to the mean phase (distances to the mean phase are marked with arrows in the second plot). This jitter is indicated in the last column. We kept only the pattern with the smallest jitter and considered the original spike train as phase-locked only if this jitter fulfilled the stringent condition (σ<0.5). In this example, the spike train is considered to be a phase-locked 1∶1 pattern. (5) We show here some examples of spike trains that were not classified as phase-locked patterns because of their too large distance or too large jitter.

In this study, our aim was to quantify the different proportions of “phase-locked spike trains” in our experimental data. Therefore, we quantified each category of q∶p patterns, where q = number of oscillation cycles and p = number of spikes. For this purpose, we used an automatic detection method to determine the limits that defined a q∶p pattern. A scheme of this method is presented in Fig. 9. Briefly, the method consisted of measuring the closeness between experimental spike trains and perfectly phase-locked theoretical q∶p patterns. In measuring this closeness, the method took into account two parameters:

1. the **relative distance**, which was based only on the difference in the number of spikes between the experimental and theoretical spike trains during each oscillation cycle;
2. the **phase jitter**, which measured how well the experimental spikes were phase-locked (based on their position in the q∶p pattern).

An experimental spike train had to fall within strict limits for these two parameters to qualify it as a “q∶p phase-locked” pattern (see the next section for additional details about those measures).

This method of detection was also used for trains produced by the simulations in Fig. 5. In this case, to optimize pattern detection and minimize random noise in the jitter, we used phase trains lasting 50 cycles (cases without noise) or 100 cycles (cases with noise) in Figs. 7 A–B, while 10-second spike trains were used in Fig. 7C–D and in Fig. 8A simplified and stricter constraint on the distance was set by measuring the firing rate, if this did not exceed 2% of the firing rate of the pattern, and a simplified constraint for jitters was set if the standard deviation of phases of all first spikes of each cycle did not exceed 0.05 cycle, which allowed us to more rapidly detect the edges between locked and unlocked zones.

#### Details of the method for pattern detection

Each spike train [observed both in the experimental results and model (described later)] was represented as a sequence of spike phases (step1, Fig. 9). From experimental observations, six types of patterns were described for the q∶p categories, which were the p spikes per q cycles (q and p are prime numbers between each other and are chosen among [1 2 3]): 3∶1, 2∶1, 1∶1, 2∶3, 1∶2, and 1∶3. In these patterns, the phases were stable from one period to the next.

Each spike train was compared with all the different types of q∶p patterns to find the closest one. This comparison occurred in two steps:

First, the distance between the spike train and a theoretical pattern was measured (step 2 Fig. 9). The **absolute distance** is the number of cycles that differ in the number of spikes. For theoretical patterns with multiple spikes per cycle, the first and last cycles could be cut to best match the experimental spike train. This absolute distance was then normalized by the number of cycles over which the spike train spread to obtain the **relative distance**, which was comparable across spike trains. Mathematically, this relative distance can be written as:
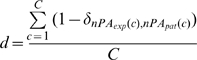
(3)where C is the total number of oscillation cycles, *nPA_exp_(c)* and *nPA_pat_(c)* are the number of spikes during the cycle c in the experimental and theoretical patterns respectively, and δ is the dirac function that equals 1 when both indices are equal.

The relative distance was computed for all types of theoretical patterns, and only the patterns that had a distance 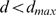 were further considered in the classification process. For all patterns, 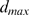 was set to 0.33, which corresponded to an allowance of up to less than one-third of the cycles where there could be excess or missing spikes relative to the theoretical pattern.

The selected set of patterns was then submitted to a second round of selection (step 3 and 4 Fig. 9) based on a criterion of phase jitter between the experimental train and each pattern. The estimator of the jitter σ is given in Eqn (4):
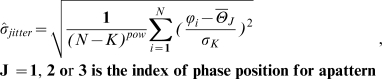
(4)where N is the total number of spikes in the train, *ϕ_i_* is the phase of each spike, 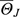 is the mean phase of the spikes that correspond to position J in the theoretical pattern cycle (thus it is different for spikes that corresponds to different positions), and *σ_K_* is a normalization factor to compensate for patterns with multiple spikes in a single cycle, with *σ_K_* equal to the standard deviation of a uniform distribution of a single spike in a 1/K cycle fraction, where K is the maximal number of spikes in the pattern cycle. The formula is slightly adapted for 2∶3 patterns: the denominator N-K takes the value N-3 in order to take into account the degree of liberty that is when phase can vary on 3 different positions and *σ_K_* changes according to the number of spike in each cycle. The parameter *pow* is equal to 1.5 and serves to adjust the formula so that it favors the detection of long trains. The theoretical pattern to be associated with the experimental train was the one with the smallest jitter among those that satisfied the distance constraints given in Eqn (1). If two theoretical patterns showed the same jitter, the one with the smallest distance was selected. The jitter limit was set to 0.5 for the phase-locked patterns. In the presented data, the spike trains that spread over at least three gamma cycles are shown, and the results were similar when spike trains spreading over at least two cycles were also examined. Some examples of residual spike trains are given in the bottom of Fig. 9.

**Figure 10:**
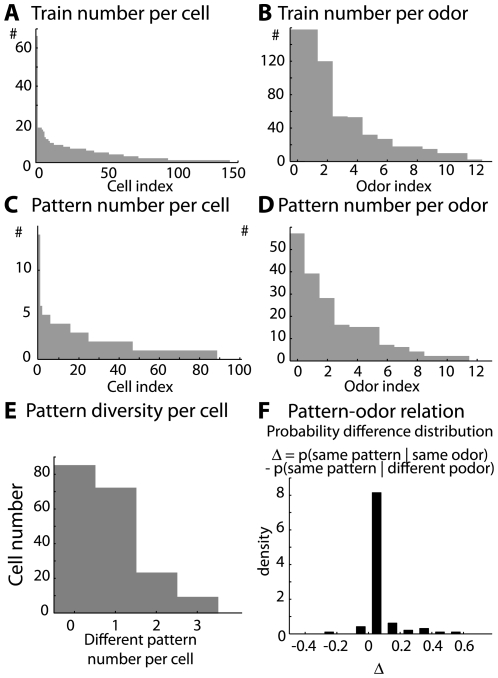
Data set information relative to cell and odors. A) Distribution of the number of spike trains per cell. B) Distribution of the number of spike trains per odor. C) Distribution of the number of detected patterns per cell. D) Distribution of the number of detected patterns per odor. In panels A–D, all of the indexes are based on a decreasing number of trains or patterns per cell or odor. E) Cell contribution to pattern variety. The majority of cells does not fire patterns, and a minority is able to evoke more than one type of pattern. F) Correlation between odor and pattern formation. The distribution of a difference of probability is plotted. The difference is estimated for each cell as (1) the probability of finding the same pattern evoked by the same odor, minus, (2) the probability of finding the same pattern evoked by different odors.

In short, our database consists of 2189 simultaneous recordings of LFP-gamma bursts that are associated with spike trains of various lengths. Other recordings where there were neither spikes nor gamma oscillations were discarded. Among the spike trains, 1210 had strictly more than 1 spike, and 898 spread over at least 3 LFP gamma oscillation cycles. These spike trains were recorded in 143 different mitral cells. Spike train distribution per cell and per odor is provided in Fig. 10A and B, respectively. The pattern study is performed on spike trains that cover at least 3 LFP gamma cycles. Detected pattern distribution over the recorded cell population and tested odor is given in Fig. 10C and D, respectively. Finally, the individual cell contribution to the detected patterns is given in Fig. 10E. The probability of finding the same pattern in an MC response was higher for responses to the same odor than for responses to different odors (see the distribution of these probability differences computed for each cell in Fig. 10F).

#### Random spike trains

To assess the significance of the phase-locked patterns, we compared our experimentally recorded spike trains to a set of computationally controlled random spike trains. Various methods can be used to draw random spike trains comparable to experimental spike trains. Since we wanted to check the influence of the oscillation on relative spike timings, it was important not to constrain neither the interspike intervals nor the spike phase relative to the oscillation, which are some measures that can be influenced by the oscillation. Therefore, we first decided to constrain our model by what appeared to be the measure that was the least contaminated by the oscillation, that is, the mean firing rate of the spike train. For each experimentally observed spike train, we drew and sorted a family of 100 spike trains using a Poisson process with the same mean firing rate but with an imposed minimal interspike interval of 1/4 of an oscillation gamma period, which corresponded to a refractory period of about 4 ms (similar to what is observed experimentally). This refractory period was reset to 0 in the few cases where firing rate of the observed spike train could not allow a refractory period as large as 4 ms. We verified that experimental and random ISI distributions were in good agreement. Each random spike train had the same number of spikes as the experimental one. Finally, the sorting of random spike trains used the same procedure as described for the experimental pattern detection. The number of spike trains generated (100 times the number of experimentally observed spike trains) was sufficient to obtain smooth distributions when compared to experimental ones.

Second, in order to see how the ISI distributions could account for the pattern proportions, instead of sorting random spike trains by the mean firing rate of their equivalent experimental spike trains, we sorted them by their global ISI distribution (see Fig. 1E). This resulted in a pattern proportion of 19%. This suggests that the ISI distribution is part of the formation of patterns. Lastly, we wanted to know whether the phase reference that is imposed by the oscillation could account for the pattern detection. We rephased each random spike train in order to make the mean phase of the spike fall in the trough of the oscillation, as this would happen under experimental conditions. When using the set of spike trains with the same ISI distribution as the experimental spike trains, we detected 25% of patterns instead of the 19% obtained without rephasing. In all cases, the proportion of phase-locked random pattern is below the proportion of experimental ones (31%), which shows that oscillations impose strong constraints on the cell discharges: the ISI distribution, phase preference in the cycle and the order of ISI occurrence.

### MC model

**Figure 11:**
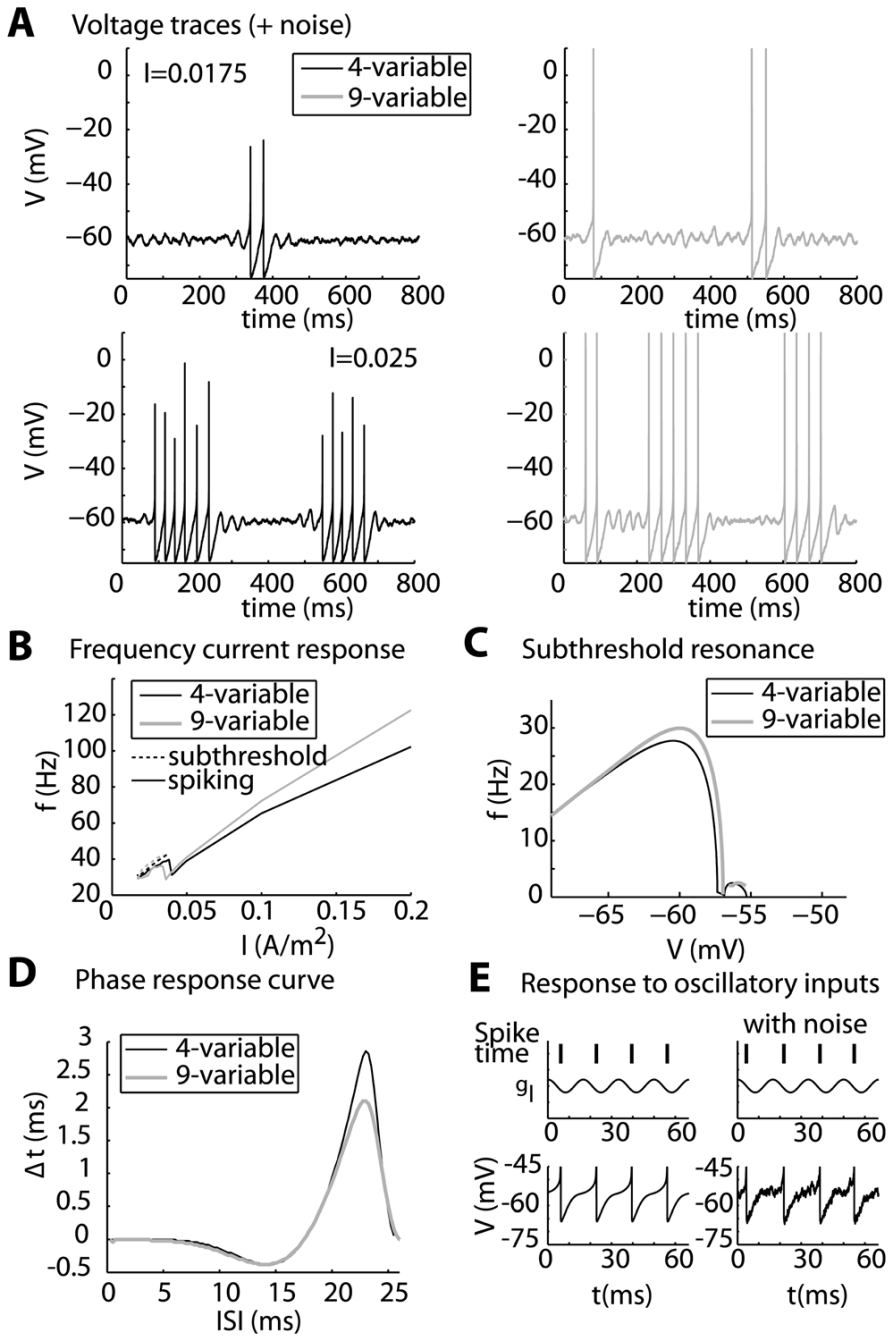
Methods for comparison of original and reduced MC model. A) Voltage traces of the original 9-variable MC model (left) and the reduced 4-variable MC model (right) are similar in terms of spike timing and subthreshold regime for similar ranges of injected current (in A/m^2^). B) Subthreshold oscillation frequency (*dashed line*) and spiking frequency (*continuous line*) are plotted as a function of the injected current for both 9-variable (*grey*) and 4-variable (*black*) models. C) Subthreshold resonance. Resonance frequency extracted from eigenvalues of the system at its resting potential for both 9-variable (*grey*) and 4-variable (*black*) models. D) Phase response curve estimated at 40 Hz spiking frequency show that integration properties are similar while both model are spiking (See method for construction). E) Response to oscillatory input. The spike times (vertical ticks), the oscillatory conductance, and the voltage trace are plotted for the 4-variable without noise (left) and with noise (right)).

The MC model was derived from a previous study [35], which was derived in turn from [85]. The model uses parameters from [86],[87]. Its essential features are (1) spiking activity through sodium spikes, (2) bursting activity (Fig. 11A), (3) its current-frequency response (Fig. 11B), (4) resonant properties as revealed through sub-threshold oscillations (Fig. 11C), and (5) phase response curve (Fig. 11D). The method to compute the resonance frequency is to linearize the system of dynamical equations around its equilibrium for a given membrane potential and to extract this frequency from the imaginary part of complex eigenvalue λ (Imag(λ)/2π). The method to compute the phase response curve [39] was to impose a small instantaneous voltage deviation along the period of spiking (here equal to 25 ms).

We reduced the number of variables in the model from nine to four by removing each of the variables individually and checking that the simpler model retained the essential features listed above; at the same time, some of the parameters were adjusted as necessary. The main characteristics were preserved as depicted in Fig. 11. The remaining variables were the membrane potential (V), the activation gating variable of the fast rectifying potassium current (m_Kf_), the activation (m_Ks_) and the inactivation (h_Ks_) gating variable of the slow potassium current. The dynamics is described in Eqs. 5–8:
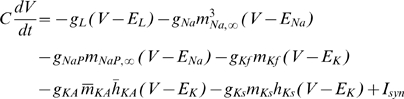
(5)

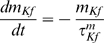
(6)

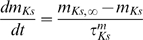
(7)

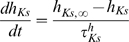
(8)I_syn_ is the synaptic current and is detailed later. The gating variable parameters are given by:
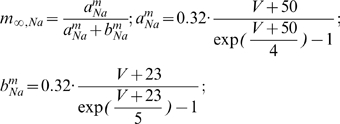


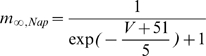


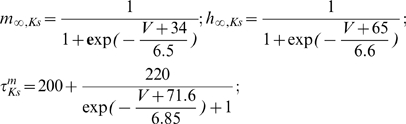


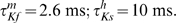
The maximal conductance of the ionic channels was g_Na_ = 500 S/m^2^; g_NaP_ = 1.1 S/m^2^; g_Ks_ = 310e S/m^2^; g_KA_ = 100 S/m^2^; g_Kf_ = 100 S/m^2^. The leak conductance is g_L_ = 0.1 S/m^2^. The product 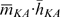 is approximated to 0.004. The reverse ionic potentials are E_K_ = −70 mV and E_Na_ = 45 mV, and the membrane reverse potential is E_L_ = −66.5 mV. The membrane capacitance is 0.01 F/cm^2^.

### Synaptic Inputs

In terms of amplitude, to assess the synaptic inputs that the MCs receive under in vivo conditions, the MC model input resistance was verified to be consistent with the order of magnitude of synaptic inputs that an MC actually receives. Experimentally, the input resistance of an MC was found to be 115 MΩ [88]. We assumed that a limited part of the total membrane area was responsible for the input resistance and that axial resistance (or intracellular resistivity) was negligible for this neuronal small portion. This portion included the soma and proximal dendrites and corresponded to 10% of the total area or ∼15.10^3^ µm^2^ [89],[90] given that, beyond a distance of about 100 µm from the soma on either the primary or secondary dendrite, synaptic inputs are not conveyed to the soma [91]. This experimental “effective” input resistance becomes 1.72 Ω.m^2^ when related to the membrane unit area. Our model is consistent with experiments since its input resistance measured at hyperpolarized membrane potential is 1.5 Ω.m^2^.

Previous studies have examined the kinetics of signaling to MCs and have found that MCs receive slow [64], fast, and asynchronous or simply fast inhibition from GCs [60]. In [17], it was reported that MCs receive weakly phased inhibition during gamma oscillation. From Fig. 4G of their report, we visually estimated that the amplitude of this phasic inhibition was up to 30%. The preferential phase of this oscillatory inhibition was estimated to be 359±9° of the LFP oscillation, and it showed strong statistical significance (p<10^−8^ by the Rayleigh test). Therefore, based on these experimental observations, our model assumes that on average, MCs receive an input current of the form (Eq.9):
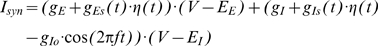
(9)which is composed of a baseline conductance, including constant excitatory (g_E_) and inhibitory (g_I_) conductances, as well as a sinusoidal part, i.e., the oscillating conductance whose amplitude is g_Io_ and whose frequency is *f_osc_*. A white noise component was added to each excitatory and inhibitory component with amplitudes g_Es_ and g_Is_, respectively, that were chosen to induce noise comparable to what is observed with in vivo intracellular voltage recording [88]. No other source of noise was added. g_Es_ and g_Is_ vary proportionally with g_E_ and g_I_ in Figs. 5 and 6. g_Es_ is set to zero in Figs. 7 and 8. g_Is_ is set to 0.282 S.m^−2^.ms^1/2^ in Fig. 7B and 0.141 S.m^−2^.ms^1/2^ in Fig. 7D.

The reversal potentials of the excitatory and inhibitory synapses are E_E_ = 0 mV and E_I_ = −70 mV, corresponding to the reversal potentials of AMPA/NMDA receptors and GABA_A_ receptors, respectively.

Inhibitory synaptic inputs can completely stop MC firing activity [50] and reshape the temporal organization of spike trains [60]. It was therefore appropriate for our model to take into account the levels of inhibitory conductance that could have such effects on MC activity. The maximum conductance of a single synaptic input has been estimated to be on the order of 1 nS [92],[93]. A single MC has around 10,000 connections with GCs. As mentioned above, only about 10% of these inputs are conveyed to the soma [91], which corresponds to ∼1,000 synapses. Such a limited integration of the total synaptic input is consistent with other studies [94],[95]. Assuming that an MC receives inhibitory inputs from 1,000 synapses during every gamma cycle (period of ∼16 ms) and assuming an exponential time decay of 5 ms for GABAa [53],[60], it can be shown that, during the gamma oscillation, the MC average inhibitory input conductance is given by 1000*1nS*5 ms/16 ms = 312 nS. For the membrane area mentioned above, this represents g_I_ = 20 S/m^2^. g_E_ was adapted to reach a frequency up to 200 Hz, as observed in vivo. For example, Fig. 11E shows a voltage trace of the MC model that is forced by the oscillation and that receives tonic excitatory and inhibitory conductances. The amplitude of the oscillatory component g_Io_ is discussed in the Results.

### Simulations

For a given input, MC activities were measured after at least one second of simulation to avoid transient effects. Euler integration was used with a time step of 0.02 ms. Simulations were performed using Matlab 7.0 (The Mathworks, www.mathworks.com).
